# Modular control of multiple pathways of *Corynebacterium glutamicum* for 5-aminolevulinic acid production

**DOI:** 10.1186/s13568-021-01335-0

**Published:** 2021-12-27

**Authors:** Fanglan Ge, Xiaokun Li, Qingrong Ge, Di Zhu, Wei Li, Fenghui Shi, Hongjin Chen

**Affiliations:** 1 College of Life Sciences, Sichuan Normal University, Chengdu, 610068 People’s Republic of China; 2 Key Laboratory for Utilization and Conservation of Bio-Resources of Education of Education Department of Sichuan Province, Chengdu, People’s Republic of China

**Keywords:** 5-aminolevulinic acid, *Corynebacteriumglutamicum*, Heme biosynthesis, Pathways engineering, Glutamate, RNA-Seq analysis

## Abstract

5-aminolevulinic acid (ALA) has broad potential applications in the medical, agricultural and food industries. Several strategies have been implemented successfully to try to improve ALA synthesis. Nonetheless, the low yield has got in the way of large-scale bio-manufacture of 5-ALA. In this study, we explored strain engineering strategies for high‐level 5‐ALA production in *Corynebacterium glutamicum* F343 using the C4 pathway. Initially, the glutamate dehydrogenase-encoding gene *gdhA* was deleted to reduce glutamate yield. Then the C4 pathway was introduced in the *gdhA* mutant strain F2-A (∆gdhA + *hemA*), resulting in a 5-ALA yield of up to 3.2 g/L. Furthermore, the accumulations of downstream metabolites such as heme, porphobilinogen, and protoporphyrin IX, were decreased. After evaluating the mechanisms of this synthetic pathway by RNA-Seq, the results showed that genes involved in both the C5 pathway and heme pathways were down-regulated in strain F2-A (∆gdhA + *hemA*). Interestingly, upstream genes of succinyl-CoA in the tricarboxylic acid (TCA) cycle, such as *icd, lpdA*, were up-regulated, while its downstream genes, including *sucC, sucD, sdhB, sdhA, sdhCD*, were down-regulated. These changes amplify the sources of succinyl-CoA and reduce its expenditure, before pulling the carbon flux to produce 5-ALA. Furthermore, the down-regulation of most genes of the heme pathway could reduce the drainage of 5‐ALA, which further enhance its accumulation. To alleviate competition between glyoxylate and the TCA cycle, the isocitrate dehydrogenase-encoding gene *aceA* was also knocked out, resulting in 3.86 g/L of 5‐ALA. Finally, the fermentation conditions were optimized, resulting in a maximum 5-ALA yield of 5.6 g/L. Overall, the blocking of the glutamate synthesis pathway could be a powerful strategy to re-allocate the carbon flux to produce 5-ALA. It could also enable the efficient synthesis of other TCA derivatives in *C. glutamicum*.

**Supplementary Information:**

The online version contains supplementary material available at 10.1186/s13568-021-01335-0.

## Key points

- Deleting gdhA sharply reduced the production of glutamate.
- Cutting down glutamate biosynthesis pathway effectively improved the 5-ALA production via the C4 pathway.
- Global transcriptomic analyses revealed the mechanism for high 5-ALA yield from the engineered *C. glutamicum* strain.

## Introduction

5-aminolevulinic acid (ALA), as a natural non-protein amino acid that occurs in living organisms, is the committed precursor for the biosynthesis of tetrapyrroles such as heme, phycobilins, chlorophyll, and cyanocobalamin (i.e., vitamin B12) (Peng et al. 1997; Kang et al. 2012), which take part in many important biological processes such as enzymatic reactions, light-harvesting and electron transport (Mobius et al. 2010). In recent decades, 5-ALA has attracted much attention because of its applications in tumor localization and cancer photodynamic therapies (Bhowmick and Girotti 2010; Inoue 2017). In addition, it has also been applied as a potential biodegradable herbicide, insecticide, or plant growth regulator in the field of agriculture (Kang et al. 2012; Hotta et al. 1997).

Naturally, two distinct endogenous routes for 5-ALA biosynthesis can be recognized. One is the C4 pathway (Shemin pathway), involving the formation of 5-ALA from succinyl-CoA (a C4-compound) and glycine via a single step decarboxylating condensation reaction, catalyzed by 5-ALA synthase (ALAS) (Warnick and Burnham 1971). This C4 pathway occurs in mammals, fungi and some photosynthetic bacteria (Kang et al. 2012; Liu et al. 2014; Li et al. 2014; Yu et al. 2020). The second route, the C5 pathway found in most bacteria and all plants, is an upstream metabolism of the heme biosynthesis (Schon et al. 1986; Sasaki et al. 2002). This pathway converts glutamate (a C5-compound) to 5‐ALA via a three‐step reaction catalyzed by the following three enzymes: glutamyl-tRNA synthetase (encoded by *gltX*), an NADPH-dependent glutamyl-tRNA reductase (encoded by *hemA*), and a glutamate-1-semialdehyde aminotransferase (encoded by *hemL*) (Kang et al. 2012). As a precursor for essential porphyrin compounds, the biosynthesis of ALA is tightly regulated by various factors such as feedback inhibition by downstream intermediates or the end product (Zhang et al. 2015). In this particular pathway, the first cyclic tetrapyrrole uroporphyrinogen III is formed from the precursor of 5- ALA in three enzymatic steps via the intermediates porphobilinogen and pre-uroporphyrinogen (Phillips 2019).Subsequently, uroporphyrinogen III is converted into heme through four consecutive enzymatic steps via the intermediates coproporphyrinogen III, protoporphyrinogen IX, and protoporphyrin IX (Phillips 2019).

Recently, great efforts had been made to construct recombinant *E. coli* and *C. glutamicum* by expressing an exogenous 5-ALA synthetase (ALAS) gene in order to improve 5-ALA production via the C4 pathway (Li et al. 2014; Yang et al. 2016; Ding et al. 2017; Chen et al. 2020; Yu et al. 2020; Miscevic et al. 2021). So far, the production of 5-ALA has been significantly improved through fermentation process optimization, pathway engineering or tolerance engineering. Similarly, the native C5 biosynthetic pathway has also been engineered in *E. coli* and *C. glutamicum* to overexpress several of the enzymes involved in order to achieve high ALA production (Liu et al. 2014; Zhang et al. 2015; Kang et al. 2011; Zhang et al. 2013; Yu et al. 2005; Zhang et al. 2020). More recently, the redistribution of carbon flux in the upstream pathway by using different promoters for driving biosynthetic genes expression and shutting down the downstream flux were also tested. For instance, to increase the available pool of succinyl-CoA via the C4 pathway, *sucCD* (encoding succinyl-CoA synthetase) was deleted (Yang et al. 2016) while *sucA* was deleted to block the TCA cycle so that carbon flux towards the ALA production via the C5 pathway could be enhanced (Noh et al. 2017).

5-ALA dehydratase (ALAD, encoded by *hemB*), catalyzing the condensation of two ALA molecules into PBG (Porphobilinogen), plays an important role in the heme pathway. In order to decrease the consumption of ALA, attempts have been made to weaken the expression of this gene, instead of completely inactivating it, as this could altogether prevent porphyrin biosynthesis (Yu et al. 2015; Zhang et al. 2019). For example, it was reported that HemB inhibitors such as levulinic acid (Sasaki et al.^1987^), D-xylose (Lin et al. 2009) and D-glucose (Lee et al. 2003) could effectively promote ALA accumulation and more recently, Zhang et al. (2019) replaced the promoter of ALA dehydratase with fliCp to weaken ALA catabolism. In a different type of approach, Su et al. (2019) fine-tuned the expression of *hemB* with CRISPR interference (CRISPRi) to improve ALA accumulation, while Miscevic et al. (2021) repressed *hemB* expression by applying CRISPRi to target various sequences in the promoter and ORF regions for achieving the same results. However, to a certain extent, the repression of *hemB* impaired the growth of cells (Miscevic et al. 2021). Similarly, the optimization of the C5 pathway not only increased the 5-ALA titer, but also resulted in the accumulation of downstream toxic compounds, such as protoporphyrin IX, which can induce changes in the overall metabolism and regulation of recombinant strains (Zhanget al. 2015; Miscevic et al. 2021).

In this work, strain engineering strategies were explored for their suitability for high‐level 5‐ALA production in *C. glutamicum* F343 via the C4 pathway. Firstly, the glutamate dehydrogenase-encoding gene *gdhA* was deleted to reduce the carbon flux to unwanted metabolites such as glutamate. Then, the *hemA* gene was introduced into the *gdhA* mutant strain to implementthe C4 pathway, with the transcription mechanisms of this synthetic pathway evaluated by RNA-Seq. In addition, the isocitrate dehydrogenase-encoding gene *aceA* was knocked out to reduce competition of glyoxylate with the TCA cycle, so that the 5‐ALA yield could be further improved. To the best of our knowledge, this is the first attempt to reduce glutamate synthesis for enhancing the 5-ALA production while shedding light on the mechanisms involved. It is expected that the results of this study will indicate that blocking the glutamate synthesis pathway can be a powerful strategy to re-allocate the carbon flux and to effectively produce many other TCA derivatives in *C. glutamicum*.

## Materials and methods

### Bacterial strains and culture conditions

Details on the bacterial strains and plasmids which were used in this study are listed in Table 1, while oligonucleotide sequences are presented in Additional file 1: Table S1. Basically, for the transformation process, *E. coli* DH5α was selected for general cloning while *Corynebacterium glutamicum* strain F343 (Zheng et al. 2012), gifted by Professor Pu Zheng of Jiangnan University, was used as the parent strain. The plasmid pK18mobsacB (Schäfer et al. 1994) was used for gene disruption in strain F343. pXMJ19 was used to express the 5-ALA synthase in *C.glutamicum*. LBHIS medium, containing 2.5 g/L yeast extract, 5 g/L tryptone, 5 g/L NaCl, 18.5 g/L brain heart infusion, and 91 g/L sorbitol was used for the electrophoretic transformation of *C. glutamicum*. The seed medium contained 20 g/L glucose, 3 g/L corn steep liquor, 5 g/L yeast extract, 10 g/L tryptone and 10 g/L NaCl. The fermentation one consisted of 35 g/L glucose, 17.1 g/L Na_2_HPO_4_·12H_2_O, 3 g/L KH_2_PO_4_; 1 g/L NH_4_Cl, 0.5 g/L NaCl, 2 g/L yeast extract, 2 g/L glycine, 1 g/L MgSO_4_·7H_2_O and 11 mg/L CaCl_2_. The fermentation medium for producing glutamate was made up of the following components: 2 g/L corn paste, 80 g/L glucose, 0.8 g/L MgSO_4_, 3.5 g/L K_2_HPO_4_, 1 g/L K_2_HPO_4_, 7 g/L urea. In addition, ampicillin (100 µg/ml), chloramphenicol (10 µg/m) as well as kanamycin (25 µg/m) were added to the medium for selection when required.

**Table 1:** List of strains and plasmids used in this study

Strain and plasmids	Genotype or description	References
Strain		
*C. glutamicum* F1	The parental strain *C.glutamicum* F343	Provided kindly by Dr. Pu Zheng, School of biotechnology, Jiangnan university (Zheng et al 2012)
*C. glutamicum* F2	*C.glutamicum* F343 ∆*gdhA*	This study
*C. glutamicum* F3	*C.glutamicum* F343 ∆*gdhA* ∆*aceA*	This study
*C. glutamicum* F1-P	*C. glutamicum* harboring pXMJ19	This study
*C. glutamicum* F1-A	*Glutamicum* harboring pXMJ19-*hemA*_*C4*_	This study
*C. glutamicum* F2-A	*C. glutamicum* ∆*gdhA* harboring pXMJ19-*hemA*_*C4*_	This study
*C. glutamicum* F3-A	*C.glutamicum* F343 ∆*gdhA* ∆*aceA* harboring pXMJ19-*hemA*_*C4*_	This study
*E. coli* DH5α	Wild-type strain; subcloning host	Lab stock
Plasmids		
pK18mobsacB	Mobilizable *E.coli* cloning vector, Km^r^	Lab stock
pK18mobsacB-∆*gdhA*	Integrative transformation vector for deletion of the *gdhA* gene	This study
pET28a( +)-hemA	pET28a ( +) carrying hemA from *R. capsulatus*	Lab stock
pXMJ19-*hemA*_*C4*_	pXMJ19 containing *hemA*_*C4*_, for overexpression	This study
pK18mobsacB-∆*aceA*	Integrative transformation vector for deletion of the *aceA* gene	This study

For 5-ALA fermentation in shake flasks, single colonies of recombinant *C*. *glutamicum* strains were first inoculated into 10 mL of the seed medium in 100-mL conical flasks and incubated at 30℃with shaking at 220 rpm for 14 h. The preculture was then inoculated into 50 mL of fermentation medium in 500-mL conical flasks to an initial OD_600_ nm of 0.5. Fermentation was performed at 30℃ and with shaking at 220 rpm, and the pH was maintained at 6.5 with 25% ammonium hydroxide throughout the fermentation. To induce gene expression, isopropyl β‐D‐1‐thiogalactopyranoside (IPTG) was added at a final concentration of 0.1 mM at 4 h of the fermentation. All shake‐flask culture experiments were performed in triplicates.

### Plasmid construction

DNA manipulation was performed according to standard protocols (Sambrook and Russell 2001) or the manufacturer’s instructions. To construct a recombinant expression vector of pXMJ19-hemA, *hemA*,encoding 5-ALA synthase from the plasmid pRSFDuet-hemA (Sambrook and Russell 2001), was amplified by polymerase chain reaction (PCR), using the primer pair Rhc hemA2 F and Rhc hemA2 R (Additional file 1: Table S1) as forward and reverse primers respectively. The PCR product was then digested with *Hin*d III and *Bam*HI, and ligated into pXMJ19 that was alsodigested using the same enzymes to produce the plasmid pXMJ19-hemA.

The primer pair gdhA-U F/R was used to amplify the upstream homologous arm of *gdhA*(encods glutamate dehydrogenase, GDH) from *C. glutamicum* F343. The PCR product was digested with *Bam*HI and *Sal*I, and ligated into the suicide vectorpK18mobsacB, digested with the same enzymes, to generate the plasmid pK18mobsacB-*gdhA*U. Similarly, after using the primer pair gdhA-D F/R to amplify the downstream homologous arm o*f gdhA* from *C. glutamicum* F343, the PCR product was digested with *Sal*I and *Hin*dIII, and ligated into pK18mobsacB-*gdhA*U that was digested with the same enzymes, to yield the plasmid pK18mobsacB-∆*gdhA*. Finally, *aceA*1 F and *aceA*1 R (Additional file 1: Table S1) were used to amplify the upstream homologous arm of *aceA*, while *aceA*2 F and *aceA*2 R were used to amplify the downstream homologous arm of *aceA*, the PCR products were inserted into pK18mobsacB, respectively, leading to plasmid pK18mobsacB-∆*aceA*.

Transformation of *C. glutamicum* F343 through electroporation was performed as described by the method of Tauch et al. (2002). Chromosomal disruption of *gdhA*, obtained via the selection of the first and second recombination events, was carried out as described by Scha¨fer et al. (Schäfer et al. 1994), and the resulting strain with *gdhA* disruption was named as strain F2. In this case, the primers Check 1 F and Check 1 R were used to check for the correct disruption of gene. Similarly, the strain *aceA* and *gdhA* double disruption was obtained and named as strain F3. Also, the primers Check 2 F and Check 2 R were used to check for the deletion of *aceA*.

### Physiological analyses

Growth was measured at 600 nm (OD_600_) using a UV–visible spectrophotometer (Shimadzu UVmini-1240, Kyoto, Japan) after diluting the culture to a proper volume with distilled water, while the concentrations of residual glucose and glutamate were determined using a glucose-glutamate analyzer SBA-40C (Biology Institute of Shandong Academy of Sciences, China). Finally, ALA concentration in the culture medium was measured as described by Mauzerall and Granick (Mauzerall and Granick 1956). Briefly, 1 mL of the supernatant of cell culture was mixed with 0.5 mL of 1 M sodium acetate (pH 4.6) and 0.25 mL of acetylacetone (2,4-pentanedione) successively, and heated at 100℃ for 15 min. After that, the samples were cooled to room temperature and modified Ehrlich's reagent (p-dimethyl aminobenzaldehyde in 95% EtOH with HClO4) was added into them. After incubation at the room temperature for 30 min, the absorbance was measured at 554 nm. For determining the concentration of PBG, 1 mL of a suitable sample diluent was mixed with 1 mL of Ehrlich’s reagent and incubated at the room temperature for 30 min, the absorbance was measured at 554 nm. The concentrations of ALA and PBG were then deduced from the standard curve prepared separately. α-ketoglutarate was determined by HPLC (Shimadzu, Japan) using a chromatographic column SB-Aq 4.6 mm × 250 mm at 40 °C as well as a mobile phase of 5 mol/L H_2_SO_4_ at a flow rate of 0.5 mL/min. The compound was detected by UV (215 nm) absorption, with an injection volume was 20 μL. The concentration of α-ketoglutarate was calculated based on a standard curve of ketoglutarate/peak area (Yu et al. 2015). Heme concentration was measured using a fluorescence-based assay, as previously reported (Sinclair et al. 2001). Briefly, a suitable sample diluent was mixed with 0.5 mL of 1 M sodium acetate at 96 °C for 30 min to remove iron from the heme. 200 µL of each sample was then transferred to a 96-well microtiter plate, after which the fluorescence was recorded on a Tecan (Tecan Trading AG, Switzerland) microplate reader (excitation at 400 nm and emission at 608 nm). The values for each sample were normalized to the emission of an unheated control sample.

Intracellular protoporphyrin IX (PPIX) concentration was determined by HPLC (Ko et al. 2018) and for this purpose, to bacteria previously collected in EP tubes, 1 ml of acetone: HCl (95:5) buffer was added. The mixture was then vortexed and diluted with 1 ml of 1 M NaOH. The intracellular sample was disrupted and supernatant was filtered using a filter membrane for concentration analysis. The porphyrin concentration was determined by high-performance liquid chromatography (HPLC) (Shimadzu, Japan), using a chromatographic column C18 4.6 mm × 150 mm at  40 °C as well as a two solvent system, with solvent A consisting of 10:90 (v/v) HPLC grade methanol:acetonitrile mixture, and solvent B containing 0.5% (v/v) trifluoroacetic acid (TFA) in HPLC grade water. A flow rate of 1 ml/min was applied for 40 min, and the absorbance was measured at 400 nm, using a linear gradient method of 20–95% solvent A in B. Eventually, the concentration of PPIX was determined based on a standard curve of PPIX/peak area.

### Transcriptomic analysis

Total RNA was extracted from *C. glutamicum* F1-P, F1-A and F2-A cultures, collected from the fermentation medium during exponential growth (32 h), using an RNeasy mini kit (QIAGEN). RNA samples were then treated with DNase I RNase Free DNase Set (QIAGEN) and Ribo-Zero rRNA Removal Kit (Epicentre Biotechnologies) to remove any genomic DNA and rRNA respectively. The resulting RNA was eventually fragmented for use as a PCR template using random primers. Strand-specific cDNA libraries were prepared with the mRNA-seq Sample Prep kit (Illumina) and the libraries were sequenced on an Illumina NovaSeq 6000 platform (Novogene, Beijing). The sequence data was deposited to the NCBI Sequence Read Archive (SRA, https://www.ncbi.nlm.nih.gov/sra) with the accession number PRJNA767542. The resulting raw reads were then cleaned by removing sequences corresponding to adapters and low-quality reads (Q ≤ 5) before mapping the clean reads to the genome of *C. glutamicum* SCgG2 (Accession number: NC_021352.1) by using the short oligonucleotide analysis package SOAP2/SOAPaligner (Li et al. 2010). Expression levels were calculated by RPKM methods (reads per kb per million reads) (Mortazavi et al. 2008), which normalizes the read counting to the gene expression level by taking the gene length and sequencing depth into account. Differentially expressed genes (DEGs) analysis was carried out on the DESeq package described by Anders and Huber (Anders and Hube 2010) while Gene Ontology (GO) enrichment analysis of the DEGs was conducted by the GO seq (Young et al. 2010). GO terms with corrected P-value < 0.05 were considered significantly enriched by DEGs.

### Statistical analysis

All experiments were conducted in triplicates. Statistical significance was determined by one-way analysis of variance, followed by Dunnett’s multiple comparison tests. Results with p values ≤ 0.05 were considered to be significant.

## Results

### Evaluation of the physiological effects of deleting gdhA, encoding glutamate dehydrogenase

Glutamate dehydrogenase (GDH), the enzyme which catalyzes the conversion of α-ketoglutarate (α-KG) and ammonium to glutamate using NAD(P)H as cofactor, links the metabolism between carbon and nitrogen (Rehm et al. 2010). For 5-ALA biosynthesis, *C. glutamicum* F343, the starting strain used in this study, harbors the native C5 pathway that uses glutamate as precursors. Hence, it was assumed that the inactivation of *gdh* might not only reduce the carbon and NAD(P)H consumption for glutamate biosynthesis, but also cut down the flux to the C5 pathway.

**Fig. 1:**
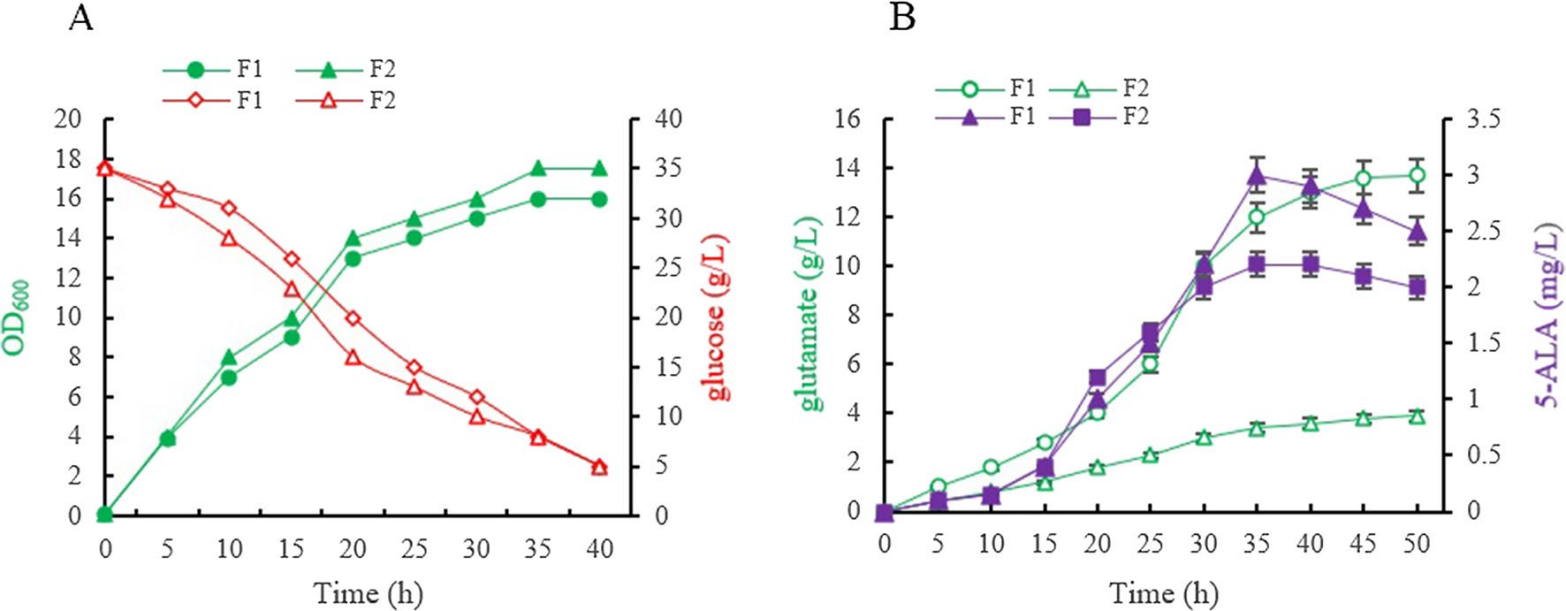
The growth and glucose consumption (**A**), the accumulation of 5-ALA and glutamate (**B**) in *C.* *glutamicum*strains F1, F2

*gdhA* (encoding GDH) was first deleted to obtain *C.glutamicum* F343 ∆*gdhA* (named as strain F2), with the latter’s physiological properties analyzed when cultivated in different media. As shown in Fig. 1A, during growth in the presence of organic nitrogen, both the starting strain (named strain F1 in this paper) and the *gdhA-*knockout mutant *s*train F2 showed similar growth rates, with F1 and F2 cells reaching an OD_600_ biomass of 16 and 18, respectively, thereby indicating that *gdhA* deletion increased the cell growth to a certain extent in a nutritious medium. On the other hand, during cultivation on CGXII minimal medium containing ammonium sulfate as the nitrogen source, cells of strain F1 and strain F2 reached an OD_600_ biomass of 0.438 and 0.12 (the initial optical density (OD_600_) for both strains are shown in Figure S1). This result suggested that *gdh* deletions significantly impaired cell growth in media containing inorganic nitrogen (e.g., ammonium).

When grown in the fermentation medium, as shown in Fig. 1B, the wild-type strain F1 accumulated 13.7 g/L of glutamate after 48 h, while the *gdhA*-knockout mutant strain F2 only accumulated 3.9 g/L of glutamate, that is, 71.5% less compared with the parent strain. This observation showed that, as expected, the deficiency of *gdhA* significantly affected the pathway involved in the conversion of α-KG to L-glutamate. Similarly, when determining the yield of 5-ALA during the fermentation process. After 36 h of incubation, the 5-ALA yield (approximately 3.0 mg/L) of the gdhA-knockout mutant strain F2 increased by 26.6% compared with the yield of the control strain F1 (2.2 mg/L) (Fig. 1B). This observation suggested that cutting down on the precursor (glutamate) could reduce 5-ALA production via the C5 pathway.

### Cutting down the pathway of glutamate synthesis to improve efficiency of 5-ALA production via the C4 pathway

5-ALA synthase (ALAS,encoded by *hemA*), which catalyzes the one-step condensation of succinyl-CoA and glycine to form ALA, is a crucial enzyme involved in the C4 pathway (Jaffe et al. 1995). Since this pathway is absent in *C. glutamicum*, the introduction of a heterologous ALAS is necessary. In our previous study, an ALAS*-*encoding *hemA* from *Rhodobacter capsulatus* was successfully cloned and expressed in *E. coli*, resulting in the 5-ALA yield being effectively improved (Ge et al. 2021). In the current study, the gene *hemA* was first inserted into pXMJ19 to produce the expression plasmid pXMJ19- hemA, that was subsequently introduced into *C. glutamicum* F343 (strain F1) and *C. glutamicum* F343∆*gdhA* (strain F2) to obtain strains F1-A and F2-A, respectively.

**Fig. 2:**
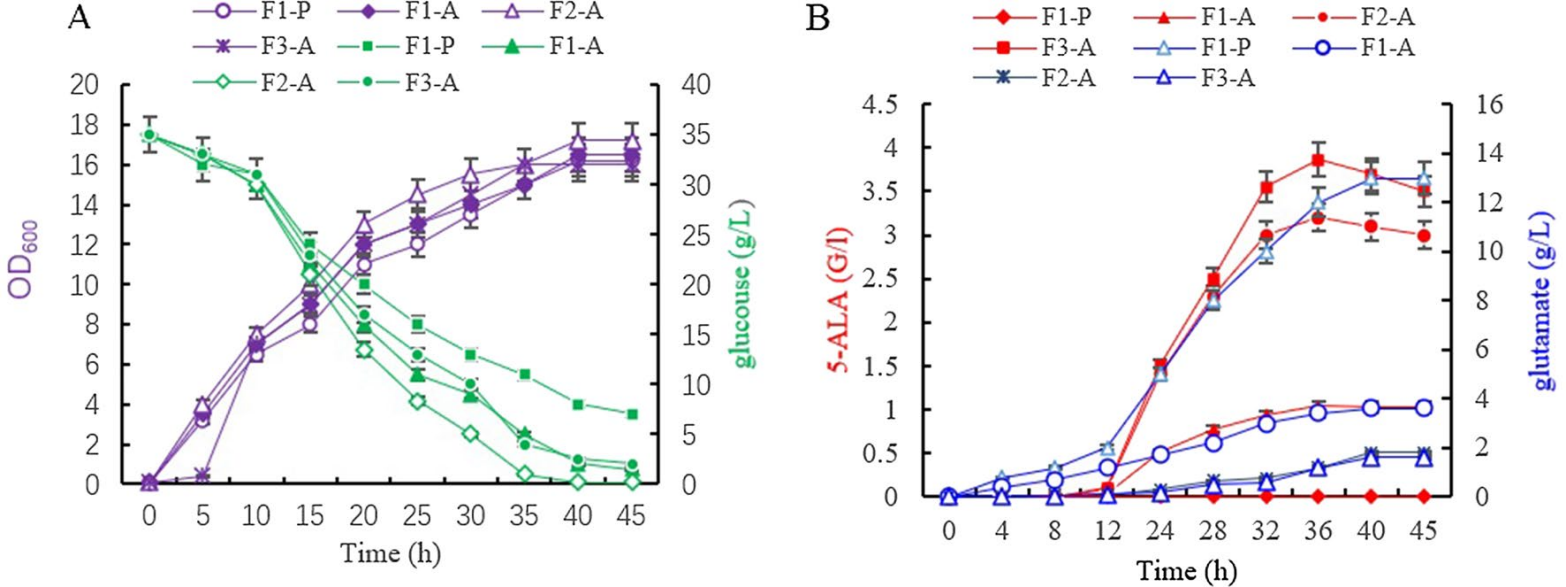
The growth and glucose consumption (**A**), the accumulation of 5-ALA and glutamate (**B**) in *C. glutamicum*strainsF1-P, F1-A, F2-A

The effects of introducing the C4 pathway on the production of glutamate were investigated during fermentation. The introduction of the gene *hemA* slightly increased the cell biomass of strainF1-A (the starting strain with pXMJ19- hemA) (OD600 of 17 at 35 h) compared with that of F1-P (the original strain with plasmid pXMJ19) (OD600 of 15 at 35 h), with both strains exhibiting similar growth rates. However, strain F1-A produced 1.1 g/L of 5-ALA after 35 h of fermentation, a value which was approximately 300-fold higher than that of the starting strain F1 (3.1 mg/L; Fig. 2C), thereby indicating that the introduction of the C4 pathway significantly improved 5-ALA production in *C. glutamicum* F343. As for strain F2-A, with inactivated *gdhA* gene, the 5-ALA production further increased (3.2 g/L) to twofold compared with strain F1-A. At the same time, strain F2-A also accumulated 1.2 g/L of glutamate, a 50% lower amount than that of strain F1-A. These results clearly supported the speculation that the inactivation of*gdhA* could pull carbon flux to the C4 pathway for 5-ALA synthesis and therefore, it was concluded that this strategy was effective for improving ALA production.

**Fig. 3:**
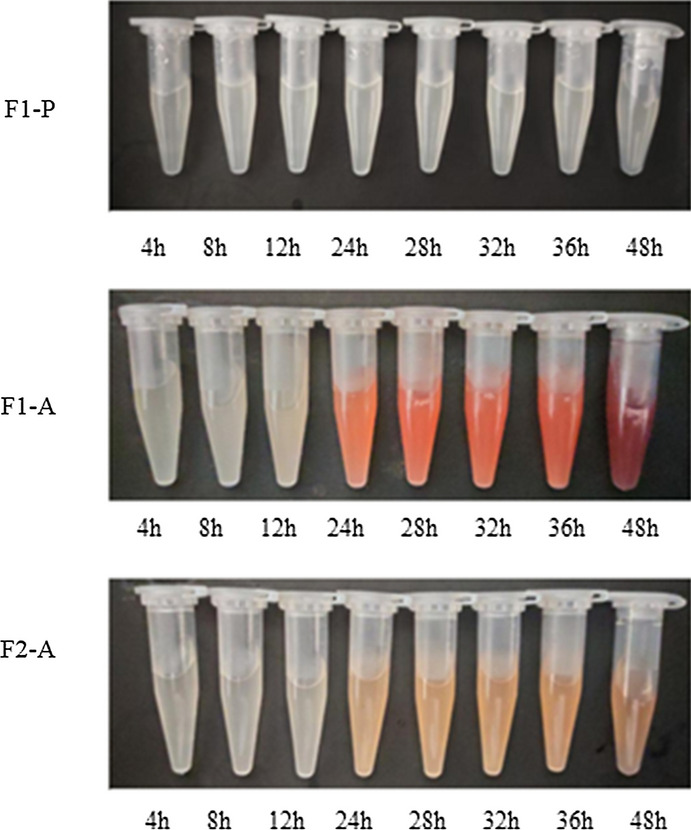
Color of fermentation broth F1-P, F1-A and F2-A

After fermentation for 24 h, strain F1-A’s (Fig. 3) culture started to turn red, and at 48 h, a dark red color, was observed, hence suggesting that porphyrin pigments were accumulated. In comparison, strain F2-A (∆gdhA + hemA), despite accumulating more ALA, exhibited a lighter color. To better understand this observation, the downstream analysis of several fermentation metabolites was carried out. In this case, after fermentation for 36 h, no porphobilinogen (PBG) and heme were detected, in strain F1-P’s culture while F1-A accumulated 263.03, 12.3, and 13.61 mg/L of PBG, PPIX and heme, respectively (Table 2). On the other hand, F2-A accumulated much less PBG (174.96 mg/l), PPIX (2.01 mg/L) and heme (9.11 mg/L) and this could explain both the higher 5-ALA content as well as the lighter color (Fig. 3). Based on the above results, it was, therefore, speculated that by decreasing the availability of glutamate, the accumulation of porphyrin pigments could be reduced.

**Table 2:** Accumulation levels of heme, PPIX, PBG in strains F1-P, F1-A and F2-A

Strain	Heme (mg/L)	PPIX (mg/L)	PBG (mg/L)
F1-P	0	0	1.914
F1-A	13.61	12.3	263.028
F2-A	9.11	2.01	174.96

### Understanding the high level of 5-ALA production in C. glutamicum strain F2-A using transcriptomics

As already pointed out, it was found that the inactivation of *gdhA*, could cut down the accumulation of glutamate and this was not only beneficial for improving 5-ALA production via the C4 pathway, but also for reducing the downstream accumulation of porphyrin pigments. In order to better understand how this synthetic pathway improved 5-ALA production,high-throughput RNA sequencing (RNA-seq) transcriptome profiling was applied to characterize the differential gene expression (DGE) between strain F1-A, strain F2-A and the control strain F1-P.

The total reads generated in each sample library ranged from 7,603,374 to 11,839,632, with a unique read match above 91% (Additional file 1: Table S2). Compared with strain F1-P, strain F1-A expressed 774 up-regulated genes and 832 down-regulated genes (Additional file 1: Table S3), suggesting that the introduction of the *hemA* altered the transcriptional profiles. Furthermore, strain F2-A showed 209 up-regulated genes and 380 down-regulated genes (Additional file 1: Table S3) compared with strain F1-A, hence indicating that the inactivation of *gdhA* also led to transcriptional changes, as previously observed in terms of the physiological changes.

**Fig. 4:**
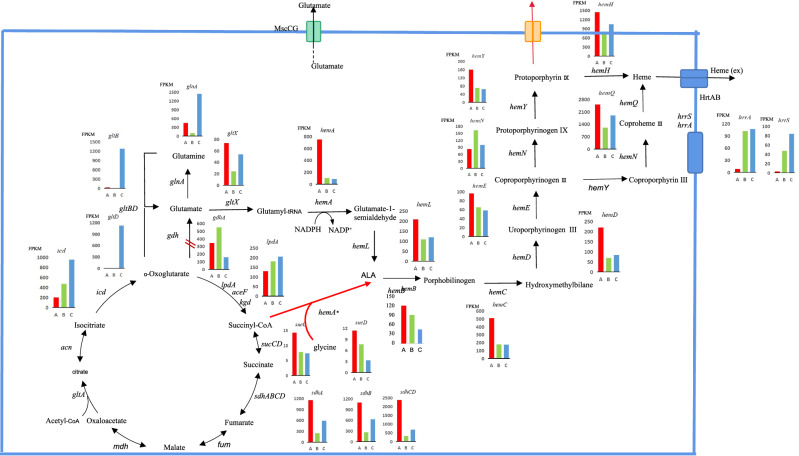
Transcription profiles of key genes involved in TCA, C5, glutamate synthesis pathway and heme pathway in *C. glutamicum* F1-P, F1-A and F2-A

Compared with strain F1-P, the transcription levels of genes involved in glycolysis, namely *pgi*, *gpmA* and *pgk*, were up-regulated in strain F1-A (Additional file 1: Table S3), and this clearly indicated that *hemA* introduction improved the utilization of glucose. Regarding the genes involved in the TCA cycle, those upstream of succinyl-CoA, including *icd* and *lpdA*, were up-regulated (Fig. 4). Interestingly, the genes downstream of succinyl-CoA, including *sucC, sucD, sdhB, sdhA, sdhCD*, were down-regulated, with these transcriptional changes favoring the supplement of precursor (succinyl-CoA) for the C4 pathway (Fig. 4). Furthermore, the introduction of *hemA* reduced the expression of genes involved in the C5 pathway. Among these, *gltx* was down-regulated 1.58-fold (log_2_ fold change), *hemA* was down-regulated 2.87-fold (log_2_ fold change), while was down-regulated 0.95-fold (Table 3) in comparison with the gene expression levels for strain F1-P. Surprisingly though, *hemA* introduction down-regulated the expression of most genes involved in the heme biosynthesis pathway (Fig. 4), except for *hemN* which was, instead, up-regulated. Such decreases in the expression levels of genes in the heme biosynthesis pathway would prevent the drainage of 5-ALA.

**Table 3:** The differentially expressed genes involved in metabolism by comparative transcriptome analysis of strains F1-P (A), F1-A (B), F2-A (C)

Gene ID	Gene name	Annotaion	Log_2_Fold change		
			BVsA	CVsA	CVsB
Genes belonging to the GS/GDH/GOGAT regulon					
*C629_RS01290*	*gltB*	Glutamate synthase large subunit	− 2.29	+ 5.53	+ 7.77
*C629_RS01295*	*gltD*	Glutamate synthase subunit beta	+ 0.10	+ 7.01	+ 6.86
*C629_RS10845*	*glnA*	Type I glutamate–ammonia ligase	− 1.98	+ 1.73	+ 3.65
*C629_RS14595*	*gdhA*	Glu dehydrogenase	+ 1.48	− 1.82	− 0.28
Genes belonging to the C5 regulon					
*C629_RS07300*	*gltX*	ABC transporter substrate-binding protein	− 1.58	− 0.42	+ 1.11
*C629_RS02480*	*hemA*	Glutamyl-tRNA reductase	− 2.87	− 3.08	− 0.26
*C629_RS02585*	*hemL*	Glutamate-1-semialdehyde	− 0.95	− 0.78	+ 0.11
Genes belonging to the TCA regulon					
*C629_RS04175*	*icd*	NADP-dependent isocitrate dehydrogenase	+ 1.22	+ 2.27	+ 0.99
*C629_RS04310*	*lpdA*	NAD(P)H-quinone dehydrogenase	+ 0.48	+ 0.69	+ 0.15
*C629_RS12810*	*sucC*	ADP-forming succinate–CoA ligase subunit beta	− 0.85	− 0.92	− 0.13
*C629_RS12805*	*sucD*	Succinate–CoA ligase subunit alpha	− 0.56	− 1.74	− 1.24
*C629_RS02275*	*sdhB*	Succinate dehydrogenase/fumarate reductase iron-sulfur subunit	− 2.04	− 0.77	+ 1.22
*C629_RS02270*	*sdhA*	Fumarate reductase/succinate dehydrogenase flavoprotein subunit	− 2.21	− 0.95	+ 1.21
*C629_RS02265*	*sdhCD*	Succinate dehydrogenase cytochrome b subunit	− 2.91	− 1.77	+ 1.08
Genes belonging to the Glycolysis					
*C629_RS05175*	*pgi*	Phosphoglucomutase	+ 1.39	+ 1.31	− 0.13
*C629_RS02375*	*gpmA*	Phosphoglyceromutase	+ 3.77	+ 3.61	− 0.21
*C629_RS08700*	*pgk*	Phosphoglycerate kinase	+ 2.07	+ 1.42	− 0.70
Genes belonging to the Heme regulon					
*C629_RS02555*	*hemB*	Porphobilinogen synthase	− 0.14	− 1.494	− 1.364
*C629_RS02485*	*hemC*	Hydroxymethylbilane synthase	− 1.50	− 1.54	− 0.09
*C629_RS02545*	*hemD*	Bifunctional uroporphyrinogen-III C-methyltransferase/uroporphyrinogen-III synthase	− 1.66	− 1.36	+ 0.24
*C629_RS02575*	*hemE*	Uroporphyrinogen decarboxylase	− 0.54	− 0.69	− 0.20
*C629_RS11225*	*hemN*	Coproporphyrinogen III oxidase	+ 0.99	+ 0.31	− 0.73
*C629_RS02580*	*hemY*	Protoporphyrinogen oxidase	− 1.16	− 1.27	− 0.16
*C629_RS09295*	*hemQ*	Chlorite dismutase family protein	− 1.04	− 0.38	+ 0.61
*C629_RS08570*	*hemH*	Ferrochelatase	− 0.84	− 0.44	+ 0.34
Two-component system					
*C629_RS14495*	*hrrA*	Two-component system, response regulator	+ 3.48	+ 3.57	+ 0.03
*C629_RS14500*	*hrrS*	Two-component system, signal transduction histidine kinase	+ 4.06	+ 4.91	+ 0.80

A: *C. glutamicum*/pXMJ19 (F1-P); B: *C. glutamicum*/pXMJ19-*hemA*_*C4*_ (F1-A); C:*C. glutamicum* ∆*gdhA*/ pXMJ19-*hemA*_*C4*_ (F2-A)

After inactivating the *gdhA* gene, the transcription levels of genes involved in nitrogen metabolism were up-regulated in strain F2-A. In this case, the transcription of *gltB*, *gltD* and *glnA* increased 7.77, 6.86 and 3.65-fold (log2 fold change), respectively (Table 3). This observation was in agreement with a previous report (Hänßler et al. 2009) whereby a *gdh* mutant elicited a partially deregulated nitrogen starvation response, in the form of increased transcription of *gltB*, *gltD* and *glnA*, thereby suggesting that strain F2-A assimilated ammonium via the GS/GOGAT pathway (Fig. 4). Compared with strain F1-A, the genes upstream of succinyl-CoA, including *icd*, and *lpdA*, were up-regulated, while those downstream of succinyl-CoA, including *sucC*, and *sucD*, were down-regulated (Fig. 4). These two features further helped to pull the carbon flux towards the C4 pathway for improving 5-ALA production. In addition, the down-regulation of *hemB*, *hemN* and *hemY* of the heme pathway in strain F2-A could reduce the drainage of 5-ALA, resulting in a lighter color as well as the accumulation of less downstream metabolites, despite a higher accumulation of 5-ALA.

### Blocking the glyoxylate cycle to further increase 5-ALA production

The glyoxylate cycle, regarded as very important for high glutamate production (Yu et al. 2005), partially overlaps the citric acid cycle. Since isocitrate lyase in the glyoxylate cycle competes with isocitrate dehydrogenase of the TCA cycle for a common substrate, we considered that deleting the *aceA* gene (encoding isocitrate dehydrogenase) could amplify the carbon flux to succinyl-CoA. Taking this into account, the gene *aceA* of strain F2-A was deleted to produce a knockout mutant strain F3-A (F343∆*aceA* ∆*gdhA*). The mutant *s*train F3-A for which both *aceA* and *gdhA* were knocked out showed a similar growth rate as the control strain F1-A (Fig. 2), with the culture reaching an approximate OD_600_ of 16. The glucose consumption rates of strain F1-P, F1-A, F2-A and F3-A were in concordance with the cell growth, hence showing that other pathways were able to make up for the loss of the glyoxylate cycle. In terms of glutamate content, strain F3-A accumulated 1.6 g/L of glutamate, which represented a 32.8% decrease compared with the parent strain F1-A (2.5 g/L) (Fig. 2), while its yield for 5-ALA reached 3.86 g/L, which represented a 20.6% increase, in contrast to F2-A. These results clearly suggested that the deletion of *aceA* further reduced the carbon flux to the glutamate biosynthesis pathway as it was the case in a previous study, which reported that glutamate production in an *aceA-*knockout mutant strain was severely impaired without affecting the cell growth (Yu et al. 2005). Overall, blocking the glyoxylate cycle was found to be beneficial to pull the carbon flux to the biosynthesis pathway of 5-ALA.

### Optimizing the culture conditions of strain F3-A for improved 5-ALA yield

**Fig. 5:**
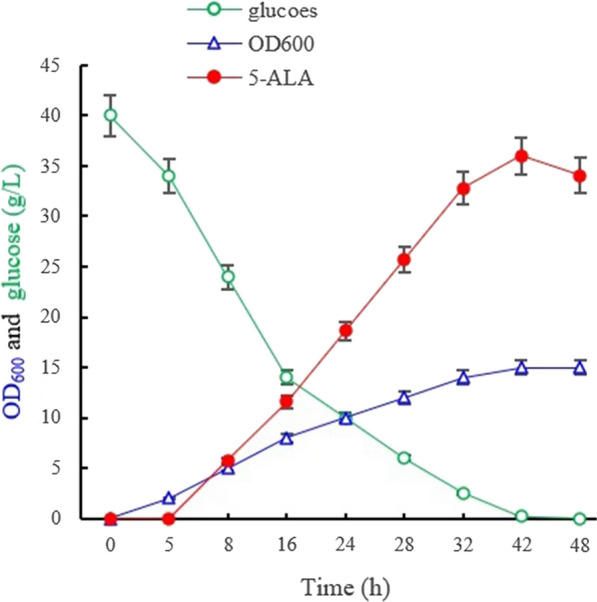
Production performance of strain F3-A in an optimal fermentation condition

To improve 5-ALA production by strain F3-A, the fermentation conditions were optimized starting with the glucose concentration. The results showed that its optimum concentration in the culture medium was 40 g/L (Fig. 3B) as with increasing glucose concentration (25, 30, 35, 40, 45 g/L) (Fig. 5), both the cell density and 5-ALA production initially fluctuated. Eventually, when the glucose concentration was at 40 g/L, 5-ALA accumulation reached its maximum (4.2 g/L) (Fig. 5). Since the expression of *hemA* can be induced by β-d-1-thiogalactopyranoside (IPTG), in order to analyze the effects of IPTG concentration on 5-ALA accumulation, 0.05, 0.1, 0.3, 0.5 and 0.7 mM of IPTG were added into the fermentation medium. The results showed that the maximum 5-ALA accumulation of 5.1 g/L was obtained when 0.5 mM of IPTG was added (Fig. 5). Finally, the effects of glycine, a precursor in the C4 pathway, on ALA production were also investigated. In the absence of glycine in the medium, 0.12 g/L of ALA was produced. On the other hand, the addition of glycine increased ALA production until a maximum amount of 5.6 g/L (Fig. 5) was reached at 8 g/L of glycine.

## Discussion

The starting strain *C. glutamicum* F343 was derived from *C. glutamicum* S9114 (Zhang and Ye 2018), a strain which is commonly used for the industrial production of glutamate. It was obvious that the high glutamate production would forcefully pull carbon flux from the TCA cycle via the reductive amination reaction of α-KG to produce glutamate, a process that consumes large amounts of organic carbon and NAD(P)H. Several researchers suggested that the deletion of the GDH-encoding gene was beneficial to improve the production of α-KG. However, other reports also showed the GDH was not required for growth and glutamate production (Börmann et al. 1993) in *C. glutamicum*. In this study, it was observed that the inactivation of the GDH-encoding gene reduced the glutamate yield sharply. In addition, as described in the previous report of Rehm et al*. *(2010), *gdhA* disruption impaired cell growth in the presence of inorganic nitrogen. However, the growth of the *gdh* mutant was free from such disruptions when cultivated in media containing organic nitrogen, hence indicating that *gdhA* was responsible for the assimilation of inorganic nitrogen. Overall, as expected, the inactivation of the GDH-encoding gene successfully reduced the carbon flux of TCA cycle to glutamate.

To enhance the accumulation of 5-ALA in *C. glutamicum*, two or more of the following strategies have been previously applied to modify *C. glutamicum*, overexpressing hemA and hemL (Yu et al. 2015; Cuiet al. 2019), down-regulating ALAD activity (Yu et al. 2015; Zhang and Ye 2018), enhancing the glycine biosynthesis pathway (Feng et al. 2016; Zou et al. 2017), expressing exporter-encoding genes (14,16), expressing codon-optimized or different sources of ALAS-encoding genes (Yang et al. 2016; Chen et al. 2020; Zou et al. 2017) and overexpressing phosphoenolpyruvate carboxylase (PPC) (16). In this work, the deletion of *gdhA* was performed to reduce the carbon flux of the TCA cycle to unwanted glutamate so that the expression of heterologous ALAS could improve the yield of 5-ALA. Furthermore, it was observed that the accumulation of downstream metabolites of 5-ALA, including PBG, PPIX and heme, were reduced, leading to a lighter color for F2-A’s culture medium.

To increase the availability of precursor succinyl-CoA, blocking of the TCA cycle by the inactivation of the *sucCD* (encoding succinyl-CoA synthetase) (Yang et al. 2016; Noh et al. 2017) or *sdhA* (encoding succinate dehydrogenase subunit A) (Miscevic et al. 2021), can be applied to enhance 5-ALA production. In this study, it was observed that inactivating the *gdhA* gene and introducing *hemA* upregulated key upstream genes while downregulating those genes upstream of succinyl-CoA (showed in Fig. 4). It is likely that this approach could increase the potential sources of succinyl-CoA and reduce its consumption, before drawing the carbon flux to produce 5-ALA. In addition, the linear molecule of 5-ALA was first converted into the cyclic compound uroporphyrinogen III via a three-step reaction, catalyzed by HemB, HemC and HemD (Fig. 4)*,* to form porphyrin pigments (Phillips 2019). Hence, the down-regulation of those three enzyme-encoding genes, as observed at transcriptional level, would not only decrease the synthesis of porphyrin and prevent the drainage of 5-ALA, but also explain the lighter color produced by strain F2-A.

## Supplementary Information

**Additional file 1: Fig. S1.** The growth a *C. glutamicum*strains F1, F2 in a medium containing ammonium as nitrogen source. **Table S1. **Primers used in this study.** Table S2. **Transcriptomic profiles of *C. glutamicum *F1-P, F1-A, F2-A.** Table S3. **Statistical results of differential genes.

## Data Availability

The data generated or analyzed during this study are included in this published article and its additional file.
