# Supplementary material for: Modular control of multiple pathways of Corynebacterium glutamicum for 5-aminolevulinic acid production
Source: AMB Express. 2021 Dec 27;11:179. doi: 10.1186/s13568-021-01335-0 (PMC8712284; doi:10.1186/s13568-021-01335-0)
Supplement: Supplementary file 1 — Additional file 1: Fig. S1. The growth a C. glutamicumstrains F1, F2 in a medium containing ammonium as nitrogen source. Table S1. Primers used in this study. Table S2. Transcriptomic profiles of C. glutamicum F1-P, F1-A, F2-A. Table S3. Statistical results of differential genes. [file 13568_2021_1335_MOESM1_ESM.pdf]

AMB Express

Modular control of multiple pathways of *Corynebacterium glutamicum* for 5-aminolevulinic acid production

Fanglan Ge*, Xiaokun Li*, Qingrong Ge*, Di Zhu, Wei Li, Fenghui Shi, Hongjin Chen

College of life Sciences, Sichuan Normal University, Chengdu, 610068, P. R. China[1]

Key Laboratory for Utilization and conservation of Bio-Resources of Education Department of Sichuan Province

*These authors contributed equally to this work.

**Corresponding author, Wei Li, liwei001@sicnu.edu.cn. Tel: 028-84480655

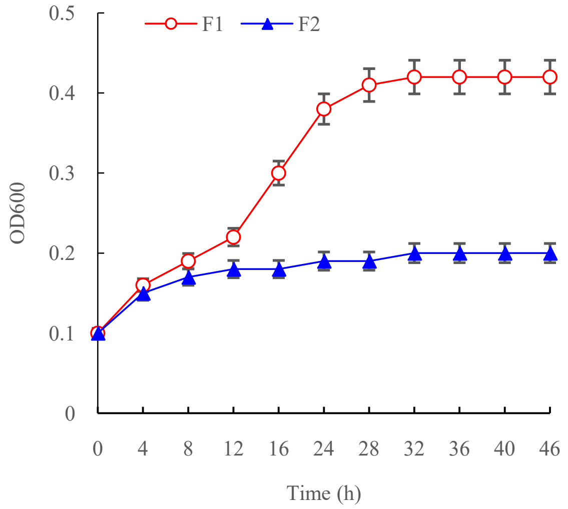

Fig. S1The growth a *C. glutamicum*strains F1, F2 in a medium containing ammonium as nitrogen source

Table S1 Primers used in this study

Primers	Sequence
*gdhA*F	CTGGAATTCGGAATCTAAGGTGTAGCTGGCTTCG
gdhA R	ACTGGATCCGAAGCCAGCTACACCTTAGATTCCG
*gdhA*F	GTGCTGCAGCAGCAGAGTATGGACACGAGAACG
gdhA R	GTCAAGCTTACAGTCACCGCAGTGCTGCTCAGTG
RhchemA F	AAGCTTAAAGGAGGACAACCATGGACTACAATCTCGCGCTCGAC
RhchemA R	GGATCCAGAATGGCTCAGGCAGAGGCC
*aceA*1 F	AGCGAATTCAGCATTCAGCGCGAAGCGTGCGTTC
*aceA*1 R	CTCGGATCCTGGATTTCCTGTGCGGTACGTGGC
*aceA*2 F	AGCTCTAGACTTCGACCAGATCGCAACCACCGTTG
*aceA*2 R	GTCAAGCTTACCTTGACGATGCGCCTTGAGTGC
Check 1 F	AGGCTGCGGCGATGTCAATGTAG
Check 1 R	CTTGAAGCCAGCAATGTTAGCGCC
Check 2 F	TTTCTGGGATGGCGTTGGTGCCGTA
Check 2 R	GTGGAACTGGCCTTCTTCAGTGGAA

Table S2 Transcriptomic profiles of *C. glutamicum*F1-P, F1-A, F2-A

Sample name	Total reads	Total mapped	Multiple mapped	Uniquely mapped
A1	8315944	8255081 (99.27%)	78575 (0.94%)	8176506 (98.32%)
A2	7789140	7731021 (99.25%)	78835 (1.01%)	7652186 (98.24%)
A3	7506966	7443287(99.15%)	85396 (1.14%)	7357891 (98.01%)
B1	7767808	7437934 (95.75%)	143952(1.85%)	7293982 (93.9%)
B2	7748094	7675343 (99.06%)	105990(1.37%)	7569353 (97.69%)
B3	7597924	7529832 (99.1%)	91194(1.2%)	7438638 (97.9%)
C1	8033310	7559076 (94.1%)	90560 (1.13%)	7468516 (92.97%)
C2	9874748	9278386 (93.96%)	122548(1.24%)	9155838 (92.72%)
C3	11513572	10567419(91.78%)	135351(1.18%)	10432068(90.61%)

A: *C. glutamicum*/pXMJ19 (F1-P); B: *C. glutamicum*/pXMJ19-*hemA*$_{C4}$(F1-A); C:*C. glutamicum*Δ*gdhA*/pXMJ19-*hemA*$_{C4}$ (F2-A)

Table S3 Statistical results of differential genes

comparation	All	Up	Down	threshold
BvsA	1606	774	832	DESeq2 padj<0.05 |log2FoldChange|>0.0
AvsC	167	91	76	DESeq2 padj<0.05 |log2FoldChange|>0.0
BvsC	589	380	209	DESeq2 padj<0.05 |log2FoldChange|>0.0

A: *C. glutamicum*/pXMJ19 (F1-P); B: *C. glutamicum*/pXMJ19-*hemA*$_{C4}$(F1-A); C:*C. glutamicum*Δ*gdhA*/pXMJ19-*hemA*$_{C4}$ (F2-A)
